# Functionalized Folic Acid-Conjugated Amphiphilic Alternating Copolymer Actively Targets 3D Multicellular Tumour Spheroids and Delivers the Hydrophobic Drug to the Inner Core

**DOI:** 10.3390/nano8080588

**Published:** 2018-08-02

**Authors:** Xia Li, Manpreet Sambi, Alexandria DeCarlo, Sergey V. Burov, Roman Akasov, Elena Markvicheva, Cecile Malardier-Jugroot, Myron R. Szewczuk

**Affiliations:** 1 Department of Chemistry and Chemical Engineering, Sawyer Mod 5, Rm 5512, Royal Military College of Canada, 11 General Crerar Crescent, Kingston, ON K7K 7B4, Canada; xiali.summer@gmail.com; 2 Department of Biomedical and Molecular Sciences, Queen’s University, Kingston, 18 Stuart St, Botterell Hall Rm 827, Kingston, ON K7L3N6, Canada; 13ms84@queensu.ca; 3 Department of Biology, Queen’s University, Kingston, ON K7L3N6, Canada; 14ald4@queensu.ca; 4 Synthesis of Peptides and Polymer Microspheres Laboratory, Institute of Macromolecular Compounds, Russian Academy of Sciences, St Petersburg 199004, Russia; burov@hq.macro.ru; 5 Department of Biomaterials and Biotechnologies, Shemyakin-Ovchinnikov Institute of Bioorganic Chemistry, Russian Academy of Sciences, Moscow 117997, Russia; zoolcat@yandex.ru (R.A.); lemarkv@hotmail.com (E.M.); 6 Institute of Molecular Medicine, Sechenov First Moscow State Medical University, Trubetskaya Str. 8-2, Moscow 119991, Russia

**Keywords:** multicellular tumour spheroids, cyclo-RGDfK(TPP) peptide, folate, amphiphilic alternating copolymer, targeted drug delivery, paclitaxel, 5-fluorouracil, curcumin, pH-responsive

## Abstract

Engineering of a “smart” drug delivery system to specifically target tumour cells has been at the forefront of cancer research, having been engineered for safer, more efficient and effective use of chemotherapy for the treatment of cancer. However, selective targeting and choosing the right cancer surface biomarker are critical for a targeted treatment to work. Currently, the available delivery systems use a two-dimensional monolayer of cancer cells to test the efficacy of the drug delivery system, but designing a “smart” drug delivery system to be specific for a tumour in vivo and to penetrate the inner core remains a major design challenge. These challenges can be overcome by using a study model that integrates the three-dimensional aspect of a tumour in a culture system. Here, we tested the efficacy of a functionalized folic acid-conjugated amphiphilic alternating copolymer poly(styrene-*alt*-maleic anhydride) (FA-DABA-SMA) via a biodegradable linker 2,4-diaminobutyric acid (DABA) to specifically target and penetrate the inner core of three-dimensional avascular human pancreatic and breast tumour spheroids in culture. The copolymer was quantitatively analyzed for its hydrophobic drug encapsulation efficiency using three different chemical drug structures with different molecular weights. Their release profiles and tumour targeting properties at various concentrations and pH environments were also characterized. Using the anticancer drug curcumin and two standard clinical chemotherapeutic hydrophobic drugs, paclitaxel and 5-fluorouracil, we tested the ability of FA-DABA-SMA nanoparticles to encapsulate the differently sized drugs and deliver them to kill monolayer pancreatic cancer cells using the WST-1 cell proliferation assay. The findings of this study revealed that the functionalized folic acid-conjugated amphiphilic alternating copolymer shows unique properties as an active “smart” tumor-targeting drug delivery system with the ability to internalize hydrophobic drugs and release the chemotherapeutics for effective killing of cancer cells. The novelty of the study is the first to demonstrate a functionalized “smart” drug delivery system encapsulated with a hydrophobic drug effectively targeting and penetrating the inner core of pancreatic and breast cancer spheroids and reducing their volumes in a dose- and time-dependent manner.

## 1. Introduction

Designing drug delivery vehicles that actively target cancer cells is an area of research interest as an alternative treatment of cancer, providing a better quality of life for cancer patients [1]. However, it is unknown how these targeted delivery vehicles interact with a three dimensional (3D) tumour mass in vivo. Although numerous delivery vehicles can selectively target cancer cells, the majority of these studies use two-dimensional cellular monolayer systems [2]. These study models do not accurately mimic the complex in vivo interactions that take place between cancer cells and their tumour microenvironment. Indeed, many delivery systems show promise in vitro, but these approaches do not similarly translate to in vivo applications. The ability of delivery drug vehicles to interact with a 3D tumour mass is initially tested in vivo and to monitor the efficacy of this interaction process in real-time is lengthy, expensive, complicated and challenging. Recent reports have investigated the application of nanoparticles to 3D tumour spheroids to characterize their ability to penetrate the mass of cancer cells [3,4]. However, these studies used nanoparticles that do not actively target overexpressed receptors on cancer cells. As such, investigating the mechanism(s) of action of targeted delivery vehicles and their interaction with 3D multicellular structures serves as an essential intermediate step between in vitro and in vivo studies.

To this end, Minchinton and Tannock [5] reported an eloquent review of the strategies to improve drug penetration through tumour mass and the design of selective compounds that have the targeted abilities to penetrate tissue [6]. It is noteworthy that 100 nm particles or larger generally do not penetrate well throughout the tumour mass, and smaller nanoparticles do not accumulate sufficiently in the tumour vasculature by the enhanced permeability and retention (EPR) effect and do not achieve good tumour penetration. Interestingly, RGD (Arg-Gly-Asp) peptides have been reported to have excellent properties in improving drug penetration into solid tumours due to their ability to interfere with the interactions between tumour cells and extracellular matrix (ECM) proteins [5,7]. In addition, other reports using αv integrin-targeting peptide, RGD-4C, showed the therapeutic efficacy in that the peptide uniquely targeted chemotherapeutic doxorubicin (Dox) to the tumour neovasculature and enhanced its inhibitory effect in human breast cancer xenografts in mice [8]. Cheresh and colleagues [9] also provided exciting data on their design of an αvβ3-targeted nanoparticle carrying Dox capable of controlling the metastatic behavior of pancreatic and renal cell cancer in mice. Importantly, they showed that this targeted delivery of Dox to the tumor vasculature revealed a 15-fold increase in the therapeutic efficacy of the drug with few or any adverse side effects.

To mimic the ECM proteins of the tumour microenvironment, Akasov et al. [10] recently reported a novel technique using a cyclo-RGDfK peptide modified with triphenylphosphonium cation (TPP), known hereafter as cyclo-RGDfK(TPP), that is capable of generating 3D multicellular spheroid structures (MCTS). The peptide can easily facilitate one-step MCTS formation and allows for the formation of tight spheroids reproducibly. The RGD (Arg-Gly-Asp) is the active motif of the extracellular protein, fibronectin, which is known to promote cell-to-cell and cell-to-matrix interactions through the interactions with α5β1 integrins. This cyclo-RGDfK(TPP) peptide method for spheroid production has been applied to several cancer types including pancreatic and breast cancer cell lines [11] and prostate cancer cell lines [12]. The spheroids obtained by this cyclo-RGDfK(TPP) peptide method allow the characterization of the penetration properties of the drug carrier and the release of the drug in the inner core of the 3D multicellular tumour spheroid structure.

**Figure 1:**
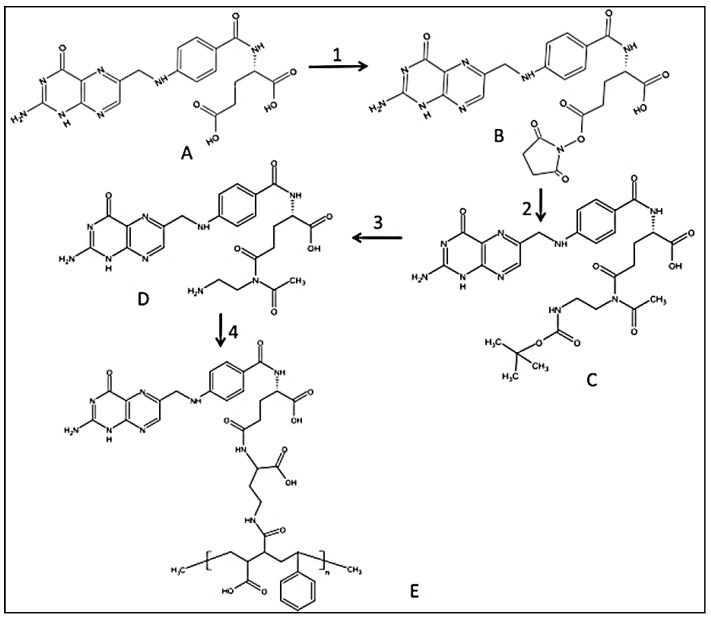
Synthetic pathway to PSMA-folic conjugates. (1) DCC/NHS Folic acid activation; (2) boc-DABA reacting with FA-NHS in DMSO, room temperature (r.t.) overnight; (3). TFA/DCM wash with TEA/DCM and precipitate with water; (4). DCC/NHS with PSMA in DMSO, r.t. overnight. Reproduced with permission from Li et al. [13].

In the present study, a “smart” functionalized folic acid biomarker was chemically conjugated to a pH-responsive, active targeting delivery system using amphiphilic alternating copolymer poly(styrene-alt-maleic anhydride) (FA-DABA-SMA) via a biodegradable linker 2,4-diaminobutyric acid (DABA) [13,14]. The rationale here is that many cancer cells overly express folic acid receptors up to two orders of magnitude in ovarian, lung, brain, head and neck, renal cell, and breast cancers [15,16]. The selection of folic acid smart biomarker allows for extensive drug delivery treatments targeting different types of cancer [17,18]. The functionalized polymer was previously characterized in detail using infrared (IR) spectroscopy, nuclear magnetic resonance (HNMR), and dynamic light scattering (DLS) confirming both the chemical structure and the pH responsiveness of folic acid-DABA-PSMA polymers [13]. The synthetic pathway of folate DABA PSMA polymer is described in Figure 1 with supplementary data on the comprehensive characterization of the polymer in the Supplementary Materials.

The carrier function of the SMA copolymer allows the formation of either amphiphilic nanotubes or nanosheets at pH 7 depending on the molecular weight of the polymer facilitating the encapsulation of hydrophobic drugs in its interior core. The self-assembly process of SMA, only observed at neutral pH, is linked to the linearity of the polymer chain, reducing the entropic cost of chain association [13]. The SMA structure is pH-responsive being stable only at neutral pH and collapses in an acidic microenvironment, releasing the hydrophobic drug on-site from its interior core. Owing to the pH-responsive nature of SMA, drugs are released once the nanoparticles reach the tumour sites with an acidic pH environment, causing the system to switch from the “off” state to the “on” state (see graphical abstract). This structural change with pH from neutral to pH 3 is reflected by a decrease in the hydrodynamic radius of the polymer from ~100 nm at pH 7 to a few nm at pH 3 [15]. The structural change results from an internal stiffening of the polymer backbone when one of the acid groups hydrolyzes at pH 7 [19]. The confined inner cavity (2.8 nm) of the nanostructure at pH7 results in an increased volume-to-surface ratio that would theoretically further increase the delivering efficiency owing to the enhanced permeability and retention (EPR) effect. The EPR effect takes advantage of the leaky vascular tumour structures with poor drainage, which would allow nanoparticles of a certain size to enter and accumulate in the tumour tissue [20].

In the present study, we investigated the efficiency of the 2.8 nm confined inner cavity of FA-DABA-SMA polymer to encapsulate the anticancer drug curcumin and two clinical standard hydrophobic chemotherapeutics, paclitaxel and 5-fluorouracil (5-FU). Each of the drugs has different complex chemical structures and molecular weights (Table 1). We examined their release and therapeutic potential on cell viability of human pancreatic PANC-1 cancer. Also, the efficacy of the smart targeting and inner core tumour penetration of FA-DABA-SMA loaded with fluorescent curcumin was investigated on pancreatic and breast cancer 3D avascular tumour spheroids, as well as its ability to reduce spheroid volumes. Currently, targeted nanoparticle-based delivery systems have not been applied with cyclo-RGDfK (TPP) mediated tumour spheroid model. The findings in this report are the first to demonstrate the novelty and therapeutic efficacy of our hydrophobic drug delivery platform and unexpectedly, provide evidence for a specific “smart” targeting of tumour spheroids resulting in the inner core penetration.

## 2. Materials and Methods

### 2.1. Cell Lines

The PANC-1 (ATCC^®^ CRL-1469™) cells are a human pancreatic ductal epithelial carcinoma cell line. The MDA-MB231 cells are a triple negative breast cancer cell line (ATCC^®^ HTB-26™). Cells were grown in media containing Dulbecco’s Modified Eagle’s Medium (1 × DMEM, Gibco, Rockville, MD, USA) supplemented in 10% fetal calf serum (HyClone, Logan, UT, USA) and 5 µg/mL Plasmocin (InvivoGen, San Diego, CA, USA) in a 5% CO_2_ incubator at 37 °C.

### 2.2. Synthesis of Folic-DABA Ligands

Folic acid is dissolved in anhydrous dimethyl sulfoxide (DMSO) and reacted with dicyclohexylcarbodiimide (DCC) and N-hydroxysuccinimide (NHS) (FA/DCC/NHS molar ratio = 1:1.2:1.2) as previously described in detail [13]. Briefly, the reaction is performed under inert nitrogen atmosphere at room temperature for 12 h. The resulting activated folate-NHS is filtered to remove *N*,*N*-dicyclohexylurea (DCU) by-product. The activated folate is dropwise added to a solution of boc-protected linker boc-2.4-diaminobutyric acid (DABA) (FA/DABA molar ratio = 1:1) in anhydrous DMSO with DCC/NHS carried out under inert nitrogen atmosphere at room temperature overnight. The aminated folate (folate-DABA-Boc) (MW = 2000) is dialysed in water and freeze-dried. The dry compound is characterized using 1H NMR in DMSO and IR via KBr pellet method. The boc protecting group is removed using trifluoroacetic acid and dichloromethane TFA/DCM at 30 °C for 6 h. TFA is evaporated under heat and vacuum, and the compound is taken up by dimethylformamide (DMF) and precipitated using ether. The final folic-DABA compound is filtered, air-dried and characterized by 1H NMR as previously described in detail [13].

### 2.3. Synthesis of Folic-DABA-Poly (Styrene-Alt-Maleic Anhydride) (PSMA) Nanoparticles

Powdered PSMA is added to the coupling agent DCC/NHS together with FA-DABA linker at a 1:10 ratio and the reaction carried out at room temperature overnight. The crude folic-DABA-PSMA is dialyzed in water for one day to remove DMSO solvent and then freeze-dried. The final FA-DABA-SMA polymers are characterized using 1H NMR and IR as previously described in detail [13] and supplemental data.

### 2.4. Curcumin Loading Capacity with SMA and FA-DABA-SMA Polymers

A standard curve of curcumin fluorescence was prepared in DMSO and measured at 520 nm. Specifically, 60 mg of curcumin powder is dissolved in 5 mL of DMSO and two-fold serial dilutions from 32,500 μM to 0.12 μM final concentrations. The fluorescence intensity was measured using Thermo Scientific Varioskan Flash Microplate Reader with 420 nm as the excitation wavelength and 520 nm as the emission wavelength. Fluorescence data were calculated as average measurements of three independent values and were plotted as a function of concentration.

Curcumin powder was loaded into SMA polymers through physical entrapment. Briefly, 1 mg of curcumin powder was added to polymer solutions at concentrations of 476 μM, 119 μM, and 95.2 μM and left on plate rocker overnight for efficient entrapment. The formulated solution (polymers loaded with curcumin) was allowed to settle overnight at room temperature. The un-dissolved curcumin was collected the following day by carefully removing the supernatant into a new tube (which was used to measure the acid-mediated release profile outlined below) without disturbing the sediment at the bottom. The precipitate was subsequently dried and re-dissolved in a 400 µL DMSO solution and vortexed. An amount of 100 μL of the dissolved precipitate was transferred in triplicate to a 96-well flat bottom UV-transparent plate and was measured using Thermo Scientific Varioskan Flash Microplate Reader. Fluorescence intensity readings were calibrated using the curcumin calibration curve to determine the respective concentration of the precipitate.

Drug loading efficiency of FA-DABA-SMA polymers was analyzed using the following equation:

(1) $$\frac{\text{Total loaded curcumin}-\text{Free curcumin}}{\text{Total curcumin loaded}}\%$$

### 2.5. Curcumin-Loaded SMA Polymers Release Profile

To measure the in vitro release of curcumin, curcumin-loaded SMA polymers were first prepared in a solution of phosphate-buffered saline (PBS) at neutral pH as described in the previous section. The supernatant was taken from the previous curcumin loading capacity section and was collected and further diluted with distilled water to 31.7 μM, 63.4 μM, and 111 μM in a final volume of 15 mL. The pH was lowered to 6.0, 5.0, and 4.0 by adding H_2_SO_4_ in a dropwise manner. It is noteworthy to mention that a strong acid was used as opposed to buffers to ensure that the precipitate that was released by the nanopolymers only contained curcumin and did not react with the buffers. For each sample, 1 mL of solution was used. At predetermined time intervals (day 1, 2, and 3), the released curcumin was collected, dried and resuspended in 400 μL of DMSO. The fluorescence intensity readings were taken by measuring the excitation at the wavelength of 420 nm and an emission wavelength of 520 nm on a Thermo Scientific Varioskan Flash Microplate Reader. The release efficiency was calculated using the following equation:

(2) $$\frac{\left(\text{Mass of curcumin released}\right)}{\left(\text{Mass of curcumin in the supernatant}\right)}\%$$

The data are presented as mean ± standard error mean (S.E.M.) based on the measurements of the samples from three replicates.

### 2.6. WST-1 Cell Proliferation Assay

The WST-1 assay was used as a measure of cell viability based on the reduction of a tetrazolium compound to a soluble derivative [21]. The absorbance that was recorded at 420 nm is directly proportional to the number of living cells in culture. At 80%–90% confluence, PANC-1 cells were added to 96-well micro-well plates at a density of 5000 cells/well and incubated overnight. The FA-DABA-SMA particles were synthesized following previously published protocols [14]. Adhered PANC-1 cells were then either exposed to increasing concentrations of SMA or FA-DABA-SMA, each with a maximum loading of the hydrophobic drugs paclitaxel and 5-FU or left untreated as controls for 24, 48, and 72 h. An amount of 100 μL of WST-1 reagent (Roche Diagnostics Division de Hoffman La Roche Limitée, Laval-des-Rapides, QC, Canada) diluted to 1:10 in culture medium was added to the wells for 2 h before the reading of absorbance at 420 nm for each time point. Cell viability was presented as a percentage of control using GraphPad Prism software. The data are presented as mean ± standard error mean (SEM) based on the measurements of the samples from three replicates.

The following formula was used to determine cell viability as a percent of control for each time point and treatment:

(3) $$\frac{\left(\left(\text{Absorbance of cells in a given concentration of drug}\right)-\left(\text{Media absorbance}\right)\right)\times 100}{\left(\text{Absorbance of cells alone}\right)-\left(\text{Media absorbance}\right)}$$

### 2.7. Generation of 3D Multicellular Tumour Spheroids

PANC1 and MDA-MB231 cells were plated at a density of 10,000 cells/well on a 96-well plate. The cells were allowed to adhere to the well surface for 3 h after which the cells were treated with 25 μM and 50 μM cyclo-RGDfK(TPP) peptide (synthesized in the laboratory of Prof S Burov, Saint-Petersburg, Russia) for PANC-1 and MDA-MB231 cells, respectively, to facilitate spheroid formation as previously described elsewhere [10]. Changes in cellular aggregation and spheroid formation were observed using inverted phase-contrast microscopy. Spheroids were defined as compact spheroids with a distinct border that contained cells which could not be distinguished from one another. In contrast, cell aggregates were defined as clusters of cells without a distinct border or rounded morphology.

### 2.8. Spheroid Volume Measurements as a Measure of Treatment Efficacy

Changes in spheroid volume measurements were used as a method to determine treatment efficacy as similar techniques used in vivo animal studies to correlated changes in tumour volume with treatment efficacy. Following complete formation of MCTS, which occurred on the 4th day, empty polymers, curcumin loaded SMA and FA-DABA-SMA, and curcumin alone were added directly to the wells at the effective concentration of 3 μM [14] for 3 days. The images were acquired with a scope-mounted camera (MA USA 02451, Thermo Fisher Scientific, Waltham, England) at 4× and 10× magnification on day 1, 2, and 3 following treatment. The radii of MCTS were calculated using a scale bar in the captured images. A minimum of 20 spheroids was measured per bar, where two separate radii measurements were taken from each spheroid to calculate the volume. The two radii measurements were averaged and used to calculate spheroid volume calculations outlined below. The following formulae, described in detail elsewhere [22], were used to calculate spheroid volumes:

10 × objective images: V = (4/3)πr^3^ (4)

where r is the average radius (microns)

4 × objective images: V = (2.5)(4/3)πr^3^ (5)

where r is the average radius (microns). For the 4 × objective images, the 2.5 parameter was used to normalize the values to the 10 × objective images.

### 2.9. Fluorescent Microscopy of Curcumin-Loaded SMA and FA-DABA-SMA Polymers Targeting 3D Multicellular Tumour Spheroids

It is noteworthy that PANC-1 cells were unable to adhere to glass coverslips and thus generating spheroids for fluorescence microscopy was not possible, and were instead conducted with MDAMB231 cells. MDAMB231 cells were plated at 35,000 cells per glass coverslip on a 24-well plate. After 3 h, the cells were treated with 50 μM cyclo-RGDfK(TPP) peptide to facilitate spheroid formation as previously described elsewhere [10]. Changes in cellular aggregation and spheroid formation were observed using inverted phase-contrast microscopy. Following complete formation of MCTS, curcumin loaded SMA and FA-DABA-SMA were added directly to wells at a concentration 3 μM. Following 0.5 h and 6 h of treatment times, coverslips were removed from the wells and mounted on slides using DAPI containing fluorescent mounting media. MCTS were analyzed with Carl Zeiss Imager 2 fluorescence microscope at 100× and 200× magnification.

### 2.10. Statistical Software

GraphPad Prism version 6.00 (La Jolla, CA, USA) for Windows was used to generate graphical representations and conduct statistical analyses of data. Comparisons between two groups from 2–3 independent experiments were made by one-way analysis of variance at 95% confidence using the unpaired *t*-test and Bonferroni’s multiple comparison tests or uncorrected Fisher’s least significant difference for comparisons between more than two groups. The SkanIt™ Software (Thermo Fisher Scientific, Waltham, England) for Windows was used to generate fluorescence intensity data.

## 3. Results and Discussion

### 3.1. Characterization of Hydrophobic Drugs Encapsulation by SMA and Release Profiles

The uptake and release profile of hydrophobic drugs by SMA copolymer were examined using curcumin, paclitaxel, and 5-FU. These drugs were individually studied for two main reasons: (a) all three drugs vary in molecular weight, and thus it was essential to ascertain the ability of our design platform to encapsulate drugs with different molecular weights and chemical structures; and (b) examine their release profiles in different pH environments. Ongoing research in our laboratory is now investigating the encapsulation capacity and release profiles of combining these hydrophobic drugs using the functionalized FA-DABA-SMA copolymer to specifically target and penetrate the inner core of 3D avascular tumour spheroids in culture.

Curcumin is initially used in these studies for its hydrophobicity as a model drug and its fluorescence properties. The encapsulation process of curcumin by our copolymer might be indicative of other therapeutic properties of hydrophobic drugs within the hydrophobic core, and its targeting effect visualization by fluorescence microscopy. Second, curcumin has a molecular weight that falls between the two hydrophobic chemotherapeutic drugs of interest in this study (Table 1), so that the encapsulation capacity of the design platform can be accurately elucidated. Third, the fluorescent property of curcumin allows for an accurate quantitative uptake profile of curcumin by the copolymer. A calibration curve was obtained with various known amounts of solubilized curcumin in DMSO recording the fluorescence intensity at 520 nm (data not included). This calibration curve was used to estimate the amount of curcumin encapsulated in SMA as well as its release profile at different pH values.

**Figure 2:**
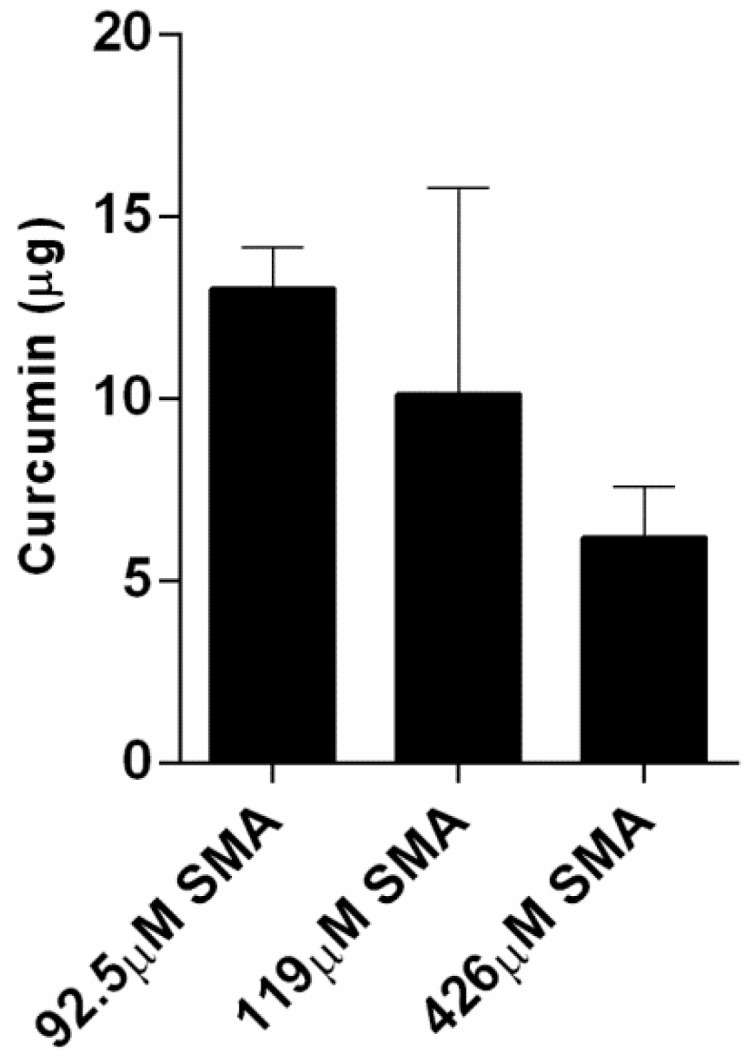
The remaining amount of the 1 mg curcumin following its encapsulation in SMA at indicated concentrations. The data represent the mean ± SEM of triplicates.

To study the uptake profile of the curcumin, three solutions of different SMA concentrations were prepared in phosphate-buffered saline, pH 7.4. An equal amount of curcumin (1 mg) was added to each concentration of the SMA in aqueous solutions, which was mixed on a rocker plate overnight to facilitate the encapsulation of the curcumin within the hydrophobic core of SMA. Following this encapsulation process, the mixture was allowed to settle overnight at room temperature for the excess insoluble curcumin to sediment. The supernatant containing the curcumin loaded SMA copolymer was then removed, and the excess sedimented curcumin was dried and dissolved in DMSO. The amount of curcumin after encapsulation is presented in Figure 2. The encapsulation efficiency was then calculated and is presented in Table 2. The results reveal a very high ratio of encapsulation for curcumin (~98%) using three different concentrations of SMA in solutions.

**Figure 3:**
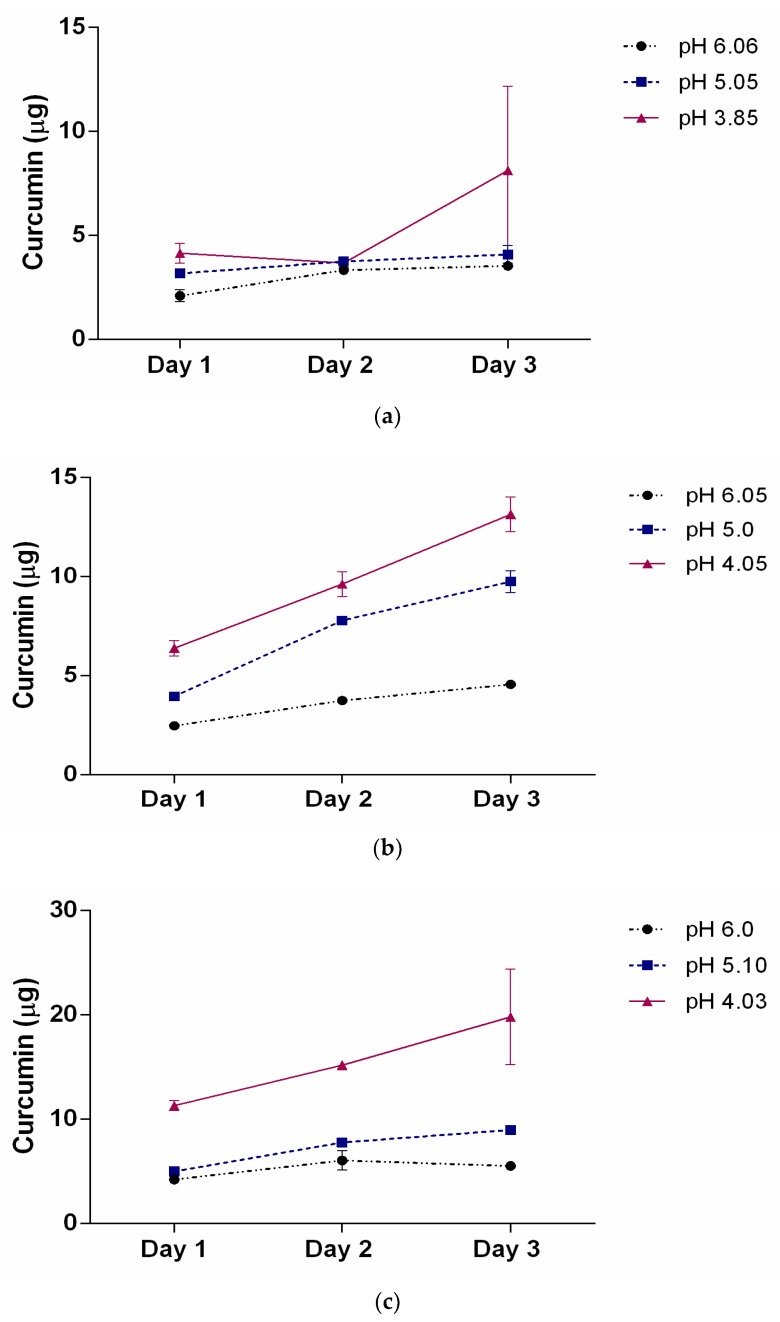
The release profile of curcumin as a function of pH over 3 days for three different SMA concentrations (**a**) 31.7 μM; (**b**) 63.4 μM, and (**c**) 111 μM. The pH of the solutions was adjusted to approximately 6, 5, and 4. The data represent the mean ± SEM of triplicates.

Table 3 illustrates the release profiles obtained for the three SMA concentrations encapsulated with curcumin after dilution in water. The curcumin loaded SMA showed viscosity which required further dilution to measure accurate release profile. The release of curcumin was monitored over three days at three different indicated pH solutions in Table 3. It is clinically relevant to measure the release profile over the course of three days as the drug released at the site of a tumour may need an extended period to be therapeutically useful. Data presented in Figure 3 reveal a slow and constant release of curcumin over three days as the pH lowers from 7.4 to 4 (approximate values).

This slow release property of SMA encapsulated curcumin could theoretically allow the gradual release of a hydrophobic drug over several days, maximally reducing cell proliferation and inducing cell death as observed previously in the case of curcumin [14]. The release efficiency at day 3 is summarized in Table 3. The data clearly show that the drug release profile is highly dependent on the pH and copolymer concentration. This pH dependency is consistent with the changes observed where the self-assembled nanotube conformation changes to random individual chains when exposed to a low pH environment.

### 3.2. SMA and FA-DABA-SMA Polymers Encapsulated with Curcumin Targeting Breast Tumour Spheroids

**Figure 4:**
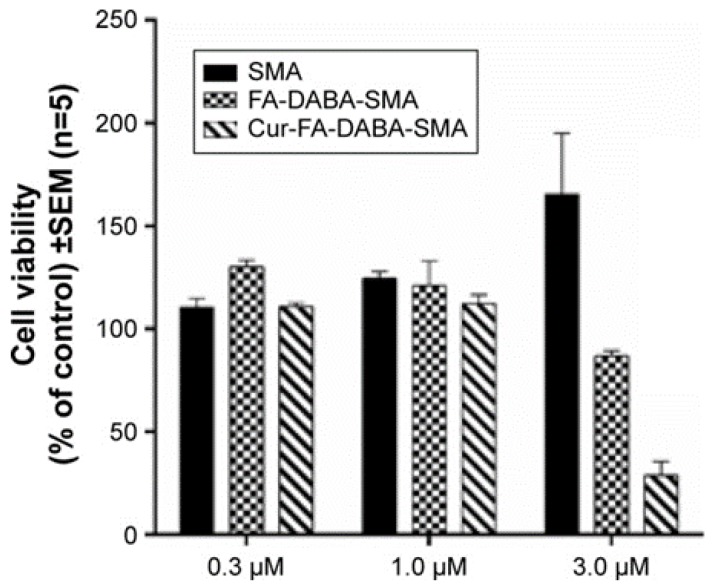
Comparison of the cell viability of PANC-1 cells at 72 h treated with SMA, FA-DABA-SMA, and Cur-encapsulated FA-DABA-SMA at different doses using the WST-1 assay (*n* = 5). Reproduced with permission from Li et al. [14].

We have previously shown the ability of FA-DABA-SMA to actively bind to the overexpressed folic acid receptors on PANC-1 cells and be internalized by receptor-mediated endocytosis [14]. Figure 4 reproduced with permission from our previous report shows curcumin-induced cell death of monolayer PANC1 cell viability assays using empty SMA and FA-DABA-SMA, and curcumin loaded FA-DABA-SMA [14]. The data indicated that when curcumin loaded FA-DABA-SMA was used at 0.3, 1, and 3 μM concentrations on PANC-1 cell viability, the results demonstrated that although the lower concentrations of curcumin (Cur)-loaded FA-DABA-SMA did not cause cell death, the 3 μM Cur/FA-DABA-SMA demonstrated significant toxicity and cell death. The toxicity at 3 μM after 72 h of treatment, compared with empty SMA and FA-DABA-SMA, confirms the on-site release of curcumin by the FA-DABA-SMA delivery platform. However, these studies were conducted on 2D monolayer PANC1 cells and did not recapitulate the tumour in a host microenvironment of cell–cell and cell–extracellular matrix (ECM) interactions that drug delivery nanoparticles would encounter in vivo.

**Figure 5:**
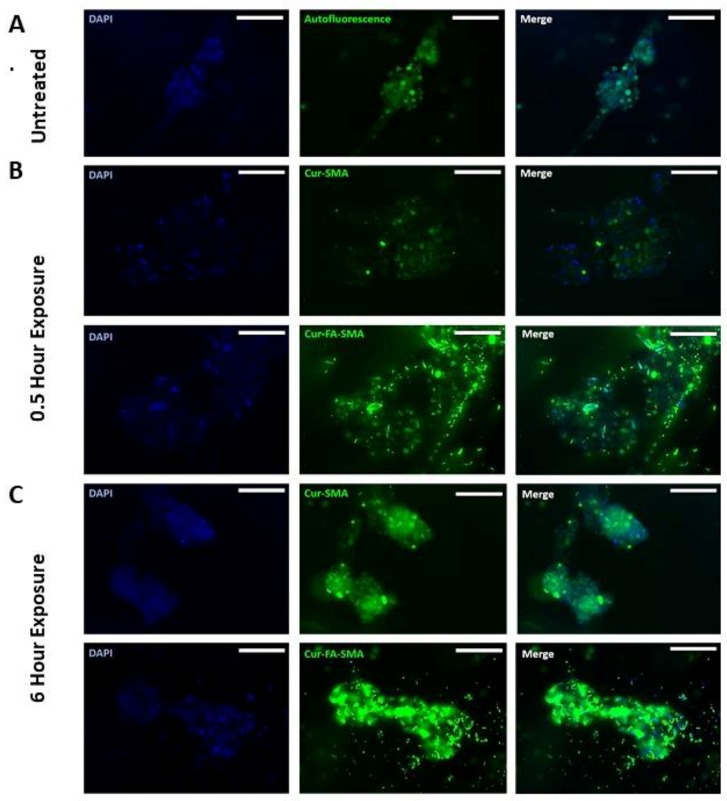
Fluorescent images at 200× objective demonstrating the variation between tumour spheroid uptake of curcumin loaded SMA and FA-DABA-SMA after 0.5 h and 6 h. (**A**) Untreated MDA-MB-231 breast cancer cell MCTS. (**B**) 3 μM Curcumin loaded SMA, and 3 μM curcumin-loaded FA-DABA-SMA was added to MDA-MB-231 spheroids for 0.5 h. (**C**) 3 μM Curcumin loaded SMA, and 3 μM curcumin-loaded FA-DABA-SMA was added to MDA-MB-231 spheroids for 6 h. The image scale bar represents 100 μm. **Abbreviations**: SMA, poly (styrene-*alt*-maleic anhydride); FA, folic acid; DABA, 2,4-diaminobutyric acid; Cur, curcumin; Auto, auto-fluorescence.

Here, breast triple-negative MDA-MB231 tumour spheroids were generated using the cyclo-RGDfK (TPP) peptide method as previously described [10,11]. It is noteworthy that PANC-1 cell aggregates do not adhere to glass coverslips, and thus were not a viable model for fluorescence studies. When spheroids were formed, the previously effective concentration of 3 μM SMA and FA-DABA-SMA loaded with curcumin [14] was tested for 0.5 and 6 h to assess the polymer’s interactions with the breast tumour spheroids. As shown in Figure 5 following 0.5 h exposure to 3 μM SMA loaded curcumin (cur-SMA) or 3 μM FA-DABA-SMA loaded curcumin (cur-FA-DABA-SMA), there was a marked difference in the uptake of curcumin by the spheroids between the SMA polymer and the functionalized FA-DABA-SMA template. The FA-DABA-SMA polymer was shown to dock on and be taken up by the tumour cells at the outer surface of the spheroids; whereas, SMA loaded curcumin polymers showed fluorescence data similar to the untreated spheroids. Following 6 h of exposure to 3 μM cur-SMA or 3 μM cur-FA-DABA-SMA, the only FA-DABA-SMA polymer revealed high penetration properties to the core of the spheroid as depicted by the decrease in the amount of visible DAPI stained cancer cell nuclei. These findings demonstrate that the functionalized FA-DABA-SMA polymer loaded hydrophobic drug effectively target 3D tumour spheroids and release their therapeutic drug cargo to the inner core of the 3D spheroid structure.

### 3.3. Changes in Tumour Spheroid Volume of PANC-1 and MDA-MB231 Cells Following Treatment with Empty and Curcumin Loaded SMA and FA-DABA-SMA Polymers

**Figure 6:**
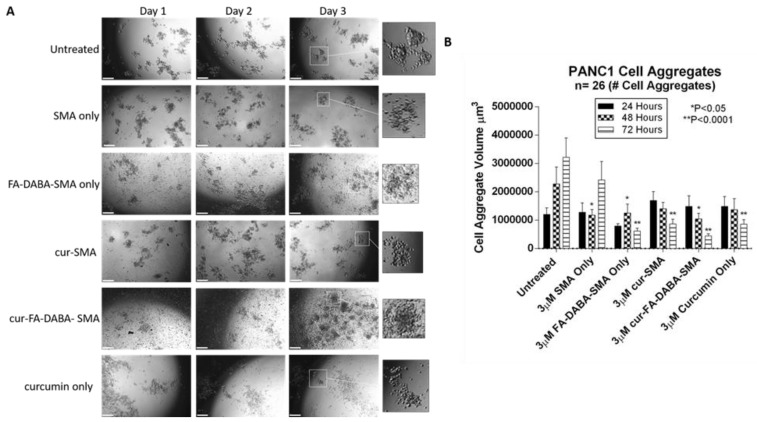
Changes in PANC-1 cell aggregate volume following exposure to 3 μM empty and curcumin loaded SMA and FA-DABA-SMA and 3 μM curcumin only. (**A**) Phase contrast images at 4× objective of changes in spheroid volume over the course of three days following exposure to 3 μM empty and curcumin loaded SMA and FA-DABA-SMA and 3 μM curcumin only. A total of 10,000 cells were plated per well in a 96 well plate for a total of 7 days (4 days for cell aggregate formation followed by 3 days of treatment). (**B**) Cell aggregate volume was measured daily following treatment using V = (4/3) πr^3^ where π = 3.1415 and r = average radius (μm). Radius was measured using a scale bar. Cell aggregate volume shown in the graph represents a cell aggregate volume ± standard error (error bars). Results were compared by a one-way ANOVA at 95% confidence using Fisher’s LSD test. The data presented in the graph are the combined results of two independent experiments that showed similar results. **Notes:** The control is represented by the spheroids that were untreated. Indicated *p*-values are a comparison between treated vs. untreated cells (*n* = 26 cell aggregates). The image scale bar represents 100 μm. The insets are magnified 300%. Phase contrast images are not a complete representation of all the spheroids that were measured and plotted in the bar graph. **Abbreviations:** SMA, poly(styrene-*alt*-maleic anhydride); FA, folic acid; DABA, 2,4-diaminobutyric acid; Cur, curcumin; CA, cell aggregate.

**Figure 7:**
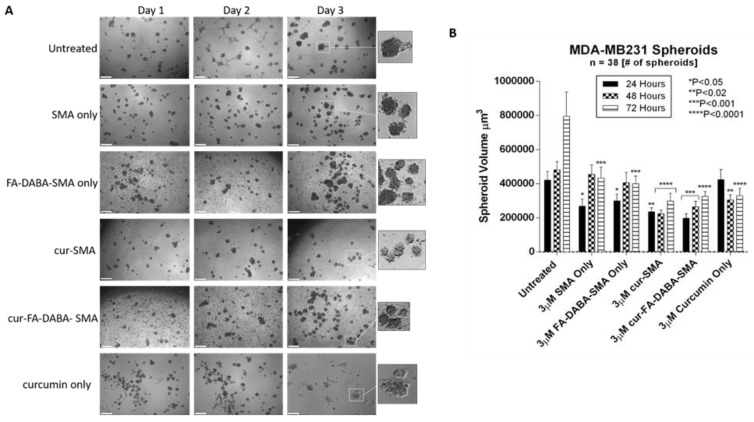
Changes in MDA-MB231 spheroid volume following exposure to 3 μM empty and curcumin loaded SMA and FA-DABA-SMA and 3 μM curcumin only. (**A**) Phase contrast images at 4× objective of changes in spheroid volume over the course of three days following exposure to 3 μM unloaded and curcumin loaded SMA and FA-DABA-SMA and 3 μM curcumin only. A total of 10,000 cells were plated per well in a 96 well plate for a total of 7 days (4 days for spheroid formation followed by 3 days of treatment). (**B**) Spheroid volume was measured daily following treatment using V = (4/3) πr^3^ where π = 3.1415 and r = average radius (μm). Radius was measured using a scale bar. Spheroid volume shown in the graph represent spheroid volume ± standard error (error bars). Results were compared by a one-way ANOVA at 95% confidence using Fisher’s LSD test. The data presented in the graph are the combined results of two independent experiments that showed similar results. **Notes:** The control is represented by the spheroids that were untreated. Indicated *p*-values vs. untreated cells (*n* = 38). The image scale bar represents 100 μm. The insets are magnified 300%. Phase contrast images are not the complete representation of all the spheroids that were measured. **Abbreviations**: SMA, poly(styrene-*alt*-maleic anhydride); FA, folic acid; DABA, 2,4-diaminobutyric acid; Cur, curcumin.

To further evaluate the anticancer effects curcumin loaded SMA and FA-DABA-SMA, changes in the spheroid volumes of PANC-1 aggregates (Figure 6) and MDA-MB231 spheroids (Figure 7) were measured and displayed. These findings revealed the biocompatibility of the polymers, as well as the cytotoxic effect of only curcumin, and curcumin loaded FA-DABA-SMA at 3 µM polymer concentrations. These results are consistent with reported observations linked to the inherent properties of curcumin suppressing the proliferation of a wide variety of cancer cells such as pancreatic, multiple myeloma, and colorectal cancer [13,23,24,25,26,27]. Also, the findings in this study using the fluorescent property of curcumin revealed that only the functionalized FA-DABA-SMA loaded curcumin is internalized through a proposed folic acid receptor-mediated endocytosis to be localized in the nucleus. A substantial reduction of cell viability was observed at 3 μM concentration for both empty FA-DABA-SMA and curcumin-loaded FA-DABA-SMA.

Since curcumin-loaded FA-DABA-SMA were able to reduce cell viability, we tested their therapeutic effects on reducing tumour spheroid volumes. Here, PANC-1 and MDA-MB231 tumour spheroids were made using a previously determined concentration of 25 μM cyclo-RGDfK (TPP) peptide. PANC-1 cells formed loose cell aggregates consistent with other reports [11], where in contrast, the MDA-MB231 cells formed compact spheroids with a distinct border and cells that were indistinguishable from one another. Both PANC1 and MDA-MB231 spheroid structures were fully formed on the 4th day following cyclo-RGDfK (TPP) peptide.

At 3 μM concentrations of empty SMA and FA-DABA-SMA polymers, 3 μM curcumin loaded SMA, 3 μM FA-DABA-SMA and 3 μM curcumin alone were added to the spheroids in culture. With 3D modelling systems, the therapeutic effects of these treatments were measured for changes in spheroid volumes similar to in vivo tumour volume measurements in humans or pre-clinical animal model of human cancer.

As shown in Figure 6, untreated PANC-1 spheroid aggregates continued to increase in spheroid volume as depicted in Figure 6B. Empty SMA showed a significant decrease in spheroid volume (*p* < 0.05) after 48 h after which time the spheroid volume continued to increase. Curcumin-loaded SMA and FA-DABA-SMA both showed a gradual decrease in cell aggregate volume. However, curcumin encapsulated FA-DABA-SMA demonstrated consistently significant decreases in spheroid volume after 48 h (*p* < 0.05) and 72 h (*p* < 0.0001) relative to untreated spheroid aggregates. In contrast, curcumin loaded SMA only demonstrated a significant decrease in spheroid volume (*p* < 0.0001) after 72 h. Unexpectedly, empty FA-DABA-SMA caused a significant decrease in spheroid volume (*p* < 0.05) after 48 h and (*p* < 0.0001) after 72 h when compared to untreated spheroid aggregates. The same observation was obtained previously when empty FA-DABA-SMA was applied to the PANC-1 cells caused a decrease in cell proliferation (Figure 5). To explain these observations, other studies have reported that the binding of folic acid to its receptor can mediate a downstream signalling cascade that is able to inhibit the cellular survival processes including cell proliferation [28,29]. Our data are consistent with these reports. Taken collectively, the therapeutic efficacy of FA-DABA-SMA loaded drug polymer may, in fact, be twofold. Binding of the folic acid on the functionalized polymer to folate acid receptors may inhibit a cellular survival activity that negatively affects tumor spheroid growth in addition to penetrating to the inner core of the tumor spheroid to unload its drug cargo. Phase contrast images showed a decrease in spheroid volume as depicted by an increase in single cells adhering to the wells in the monolayers and detaching from the cell aggregates. Similarly, untreated MDA-MB231 spheroids also continued to increase in spheroid volume as indicated in Figure 7B. The MDA-MB231 spheroids showed a spheroid volume decrease following treatment with the polymers, similarly to those results with PANC-1 spheroid aggregates. Surprisingly, both curcumin encapsulated SMA and FA-DABA-SMA showed a similar decrease in spheroid volume when compared with untreated spheroids.

One possible explanation is that the tight MDA-MB231 spheroid structure may render them resistant to the anticancer effects of curcumin. A similar finding was reported by Akasov et al. [10] where breast cancer spheroids generated from MCF-7 cells were more resistant to the cytotoxic effects of curcumin when compared to monolayer cells. The findings in this report describe the efficacy of this unique delivery system in the treatment of pancreatic and breast tumour spheroids and its ability to disable the survival mechanism(s) of tumours with acquired chemoresistance.

### 3.4. The Effects of Clinical Standard Hydrophobic Chemotherapeutics Drug Loaded Functionalized FA-DABA-SMA Polymers on Cell Viability Using Monolayer Cancer Cells

**Figure 8:**
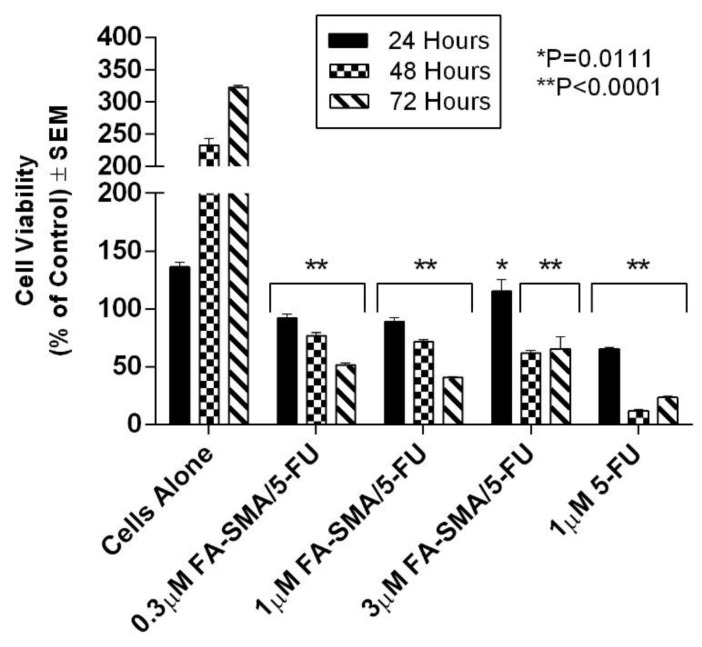
Cell viability of PANC-1 cells at 72 h treated with 0.3 μM FA-DABA-SMA/5-FU, 1.0 μM FA-DABA-SMA/5-FU, 3.0 μM FA-DABA-SMA/5-FU, and 1.0 μM of 5-FU using the WST-1 assay. 5-FU was encapsulated at maximum loading capacity in each sample. The data represent the mean ± SEM of *n* = 5 independent experiments.

**Figure 9:**
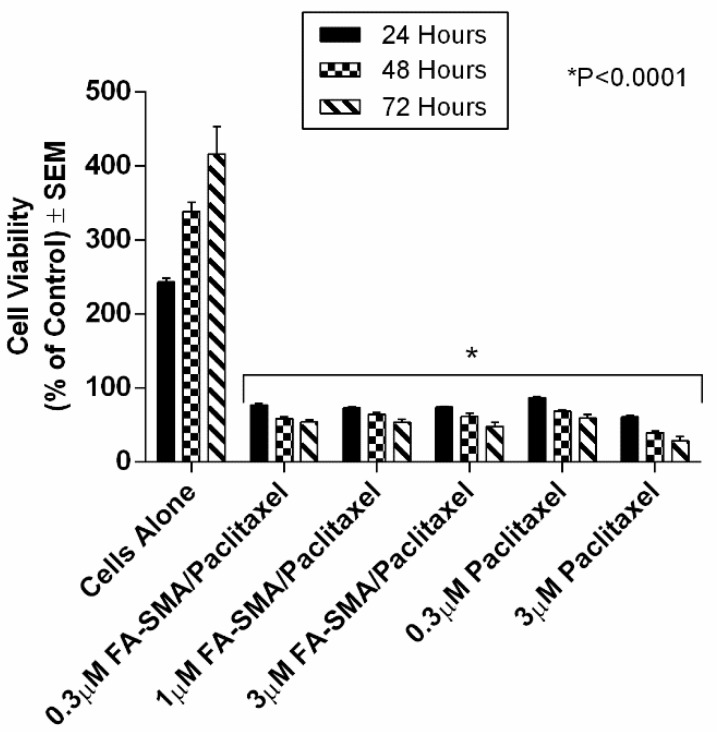
Comparison of the viability of PANC-1 cells at 72 h treated with 0.3 μM FA-SMA/Paclitaxel, 1.0 μM FA-DABA-SMA/Paclitaxel, 3.0 μM FA-SMA/Paclitaxel, 0.3 μM of Paclitaxel, and 3.0 μM of Paclitaxel using the WST-1 assay. Paclitaxel was encapsulated at maximum loading capacity in each sample. The data represent the mean ± SEM of *n* = 5 independent experiments.

The functionalized FA-DABA-SMA polymers encapsulated with clinically standard chemotherapeutics 5-FU and paclitaxel were also examined for the therapeutic effectiveness following their release from the polymer’s hydrophobic core using monolayer of PANC1 cells in culture. The therapeutic effects of the polymer released 5-FU and paclitaxel on PANC1 cell viability are presented in Figure 8 and Figure 9. Significant suppression of cell proliferation is observed in Figure 8 at low concentrations of the drugs. The decrease over time is also indicative of a slow release of the drugs over 72 h, which is consistent with results presented in Table 3. The most significant trend highlighted in the cell viability study is the very similar effect obtained at different concentrations of FA-DABA-SMA loaded with 5-FU as well as 5-FU directly in contact with the cells (Figure 8). Based on the release profile obtained with curcumin, the concentration of 5-FU released from the 1.0 μM FA-DABA-SMA loaded 5-FU is estimated at a maximum of 0.83 μM, and it has the same effect as 1.0 μM 5-FU in direct contact with the cells. This similar trend reveals the active and sustained release of the encapsulated drug on the cancer cells over time. Indeed, the injection concentration needed for direct delivery of a hydrophobic drug would be much higher to obtain a sufficient concentration of 1.0 μM to the cancerous cell to be treated. Additionally, a significant clinical challenge in treating cancer patients is the cardiotoxic effects of 5-FU chemotherapy, and to overcome this adverse effect; it is proposed that drug delivery vehicles directly target cancer cells [30]. The data depicted in Figure 8 demonstrate that the functionalized FA-DABA-SMA loaded drug polymers are not only able to bind to folic acid receptors that are overexpressed on cancer cells, but are also able to exert cytotoxic effects with an equal significance when compared with a drug directly applied to the cells in culture. In contrast, other reports have shown the release of a drug by an amphiphilic polymer carrier capable of self-assembly often results in lower effectiveness of the drug in the reduction of cell proliferation compared to the drug alone directly in contact with the cell [31,32,33]. This decrease in effectiveness can translate to a reduction of up to 50% in cell death [31]. The functionalized FA-DABA-SMA loaded hydrophobic drug polymers showed a slow release profile over 3 days avoiding the initial burst release and allowing for a more extended treatment window and potential reduction in side effects.

A similar trend in reducing cell viability by FA-DABA-SMA loaded with paclitaxel is observed in Figure 9. The large and complex chemical structure of the hydrophobic paclitaxel molecule released from the polymer efficiently suppressed the growth of the PANC1 cells even at low concentrations. Also, the slow release observed for the paclitaxel is consistent with the slow release profile observed for curcumin previously reported in Table 3 and Figure 8. With paclitaxel, there is clear evidence for the gradual and consistent decrease in cell viability over time. Surprisingly, the different concentrations of the FA-DABA-SMA polymer encapsulated with the indicated chemotherapeutic drugs had a significant effect on the cell viability and suggested that a constant release of the drug could be prolonged at higher concentrations.

The high therapeutic effects observed for the delivery of hydrophobic drugs with various molecular weights and different chemical structures implies the chemical design of the FA-DABA-SMA delivery platform releases drugs efficiently and slowly with no loss of activity. Similarly, the subsequent decrease in cell proliferation at low drug concentrations would suggest a direct internalization of the drug to the nucleus. While the release of curcumin with SMA as a carrier was observed in the cytoplasm, fluorescence microscopy demonstrated that the FA-DABA-SMA polymer encapsulated with curcumin was internalized and targeted to the nucleus leading to cell death [14]. Boshnjaku et al. [34] recently reported on the nuclear localization of the folate receptor and its novel activity as a transcription factor. The report showed that folate receptor alpha (FRα) translocates to the nucleus and interacts with promoter regions of genes such as *Fgfr4* to regulate their expression. Therefore, direct access of the drug to the nucleus may be associated with the interaction between the folate-conjugated polymer and the folic acid receptors of the cells triggering receptor-mediated internalization.

## 4. Conclusions

A novel approach to study the effects of a “smart” drug delivery system on 3D multicellular PANC-1 cell spheroid aggregates and breast tumour spheroids was investigated to resemble the interactions that would likely occur in vivo. For the first time, folic acid functionalized SMA polymers could interact with breast tumour spheroids and deliver encapsulated curcumin time-dependently to the inner core of the spheroid. Additionally, tumour volume measurements have shown that FA-DABA-SMA encapsulated with hydrophobic drugs is the far more effective delivery system when compared with SMA as it shows a gradual, yet consistently significant decrease in spheroid volume over the course of three days. A surprising observation from this study revealed that the empty FA-DABA-SMA polymers are capable of reducing spheroid volume, which suggests that the folic acid functionalized polymer may exert its therapeutic effects in combinatorial and sequential delivery effects, independently but yet, in a significant manner. The binding of folic acid to its receptor may inhibit cell proliferation while receptor-mediated internalization may allow direct interaction between the drug and the nucleus leading to cancer cell death. Additionally, the influence on cell viability by the folic acid functionalized polymer may be attributed to the novel mechanism of action of the FRα whereby the receptor acts as a transcription factor that localizes to the nucleus to regulate gene expression [34]. It is possible that a reduction in spheroid volume following treatment with empty FA-DABA-SMA results from an unknown mechanism of action mediated through folic acid binding to its receptor.

In the present study, curcumin was used for its hydrophobicity properties but also, it acts as a fluorescent probe for tracing drug uptake in our FA-DABA-SMA copolymer in the spheroid studies. The data depicted in Figure 4 and reported by Li et al. [14] demonstrate that the lower concentrations of curcumin-loaded FA-DABA-SMA did not cause cell death but instead, the 3 μM curcumin/FA-DABA-SMA had significant toxicity and cell death. Importantly, the toxicity effects of curcumin/FA-DABA-SMA at 3 μM after 72 h of treatment of cancer cells, compared with empty SMA and FA-DABA-SMA, confirms the on-site release of curcumin by the FA-DABA-SMA delivery platform [14]. Turmeric is the main source of curcumin. Chemically, curcumin ((1,7-bis(4-hydroxy-3-methoxyphenyl)-1,6-heptadiene-3,5-dione), is also called diferuloylmethane and is the primary natural polyphenol found in the rhizome of *Curcuma longa* (turmeric) [35]. However, the exact mechanism(s) of action and its bioactive components have only recently been investigated and eloquently reviewed [36]. Curcumin can target multiple signalling molecules thereby providing multiple health benefits [36]. Most of these benefits are due to its antioxidant and anti-inflammatory effects [37]. Potential therapeutic effects of curcumin as an anti-inflammatory agent, include neurodegenerative, cardiovascular, pulmonary, metabolic, autoimmune and neoplastic diseases [38]. Also, curcumin has been reported to induce apoptosis in human colorectal carcinoma by regulating expression of Prp4 and p53 [25] or through regulation of the function of MDR1 and reactive oxygen species [24]. 

For effective targeting of solid tumours, many of the chemotherapeutic drugs originating from the neovasculature tumour blood vessels can only penetrate three to five cells, and this interaction has been proposed for their limited efficacy with the subsequent development of chemoresistance [6]. Thus, the therapeutic efficacy of anticancer drugs is limited to their poor penetration into the tumour mass in addition to their adverse side effects on healthy cells, which limit the dosage of the drug that can be administered safely to patients. To improve the tumour drug penetration, Sugahara et al. [7] identified a tumour penetrating peptide which enhances the efficacy of anticancer drugs by targeting αvβ3 specifically expressed on the endothelium of tumour vessels. Here, they designed a tumour penetrating peptide, iRGD (CRGDK/RGPD/EC), chemically conjugated to a chemotherapeutic drug which facilitated the penetration of the drug deep into the tumour mass by binding to αv integrins. The report further discloses the mechanism of iRGD facilitated penetration into the tumour mass. iRGD is proteolytically cleaved in the tumour mass to produce CRGDK/R truncated peptide. This truncated peptide dissociates from its initial interaction with the integrins and preferentially binds to neuropilin-1 (NRP-1). The CRGDK/R truncated peptide, and NRP-1 complex with co-administration of anticancer drug facilitates tumour mass penetration.

**Figure 10:**
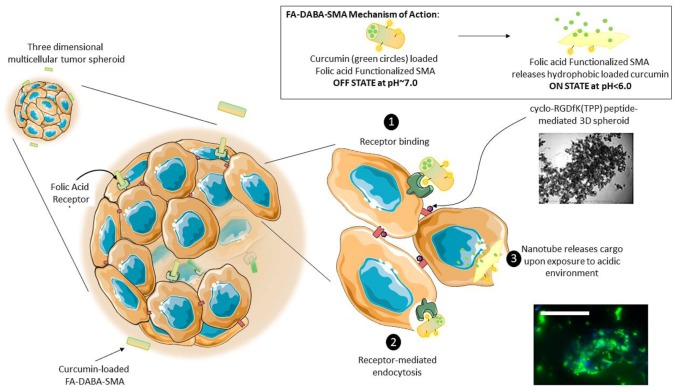
The proposed mechanism of nanoparticle transport and penetration through 3D tumour spheroid mass. FA-SMA loaded curcumin nanoparticles are transported through tumour spheroid mass by free diffusion in extracellular space. Some nanoparticles can involve cell binding to folic acid receptors and go through cell internalization or change their conformation into individual chains thereby effectively releasing their cargo. This schematic illustrates a pH-responsive, “smart” active polymeric delivery system using folate functionalized amphiphilic alternating copolymer poly(styrene-alt-maleic anhydride) (FA-DABA-SMA) via a biodegradable linker 2,4-diaminobutyric acid (DABA). The “off state” occurs at a neutral pH when the polymer self-assembles into an ordered nanotube conformation (as depicted in the top right panel) and is capable of encapsulating a hydrophobic agent, such as curcumin as pictured above. In an acidic tumour microenvironment, these nanotubes change their conformation into individual chains thereby effectively releasing their cargo. These curcumin loaded nanotubes are proposed to interact with 3D tumour spheroids through FA-DABA-SMA binding to the overexpressed folic acid receptors on pancreatic cancer cells (1) and are internalized through receptor-mediated endocytosis (2). The hydrophobic drug is then released intracellularly (3). While the cancer cells adhere to each other to generate a spheroid, the nanotubes containing curcumin are capable of penetrating the inner of a spheroid and delivering the therapeutic agent as shown in the fluorescence image. Scale bar represents 100 μm.

In the present study, the 3D spheroid model using cyclo-RGDfK (TPP) peptide may be better suited for in vitro cancer research. Akasov et al. [10] showed that the 3D spheroid formation by using synthetic cyclo-RGDfK (TPP) peptide closely mimics the natural ECM proteins binding to α5β1 integrins on the cell membrane [10]. The cyclo-RGDfK (TPP) peptide promotes the self-assembly of cancer cells reproducibly in one single step. The mechanism of FA-SMA loaded curcumin nanoparticles transported through tumour spheroid mass by free diffusion in extracellular space may be facilitated by cyclo-RGDfK (TPP) and α5β1 integrin interactions as depicted in Figure 10 and described by Sugahara et al. [7]. In Figure 10, other FA-DABA-SMA nanoparticles can involve cell binding to folic acid receptors and go through cellular internalization or change their conformation into individual chains via pH change, thereby effectively releasing their cargo. It is noteworthy that when the uptake of folate-conjugated liposomes was compared between folate receptor expressing tumours and folate receptor plus inflammatory lesions, both folate-targeted and non-targeted liposomes accumulated more readily at sites of inflammation than in solid tumours [39]. RGD-conjugated nanoparticles might hold promise for chemo drug delivery to solid tumours through their ability to interfere with integrin-ECM interactions. The therapeutic potential can enable penetration into solid tumours, by interfering directly with tumour neovasculature and integrin-mediated growth signalling by cancer cells [5].

In conclusion, pH-sensitive and biocompatible functionalized folic acid conjugated SMA copolymers are excellent carriers for the delivery of hydrophobic therapeutic drugs in a safe, effective and controlled manner. This delivery system can penetrate the inner core of breast tumour spheroids in a time- and dose-dependent manner. These unique properties of the folate-conjugated amphiphilic alternating copolymer may provide promising clinical applications, particularly overcoming the adverse cytotoxic side effects of chemotherapeutics on healthy cells.

## Figures and Tables

**Table 1:** List of drugs and model drug used in this study.

Name	Chemical Structure	Molecular Weight
Curcumin	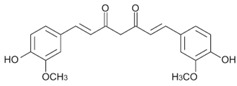	368.4 g·mol^−1^
5-Fluorouracil (5-FU)	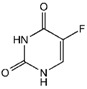	130.1 g·mol^−1^
Paclitaxel	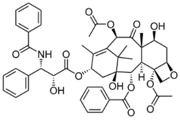	853.9 g·mol^−1^

**Table 2:** The curcumin encapsulation efficiency of three different concentrations of SMA.

SMA Polymer Concentration	Curcumin Precipitation	Encapsulation Efficiency
92.5 μM	13 μg	98.7%
119.0 μM	11 μg	98.9%
426.0 μM	6 μg	99.4%

**Table 3:** Release efficiency of curcumin at day 3 at pH 4, 5, and 6 of a final concentration of 31.7, 63.4, and 111 μM of SMA diluted in water from initial SMA concentrations in Figure 2 and Table 2.

Concentration of SMA	pH 4		pH 5		pH 6	
	Mass	% Released	Mass	% Released	Mass	% Released
31.7 μM	6.14 μg	7.0%	3.68 μg	5.5%	3.47 μg	5.27%
63.4 μM	13.33 μg	20.1%	10.22 μg	15.4%	5.11 μg	7.7%
111 μM	17.53 μg	26.6%	8.79 μg	13.3%	5.30 μg	8%

## Supplementary Materials

The following are available online at http://www.mdpi.com/2079-4991/8/8/588/s1, Figure S1: Two different conformations of the quadrimers of SMA at pH 7 corresponding to two chiralities (SR−RR−SR and SR−SR−SR). The structures are very linear, and the orientations of the benzene groups are similar. Reproduced with permission from [S1], Figure S2: Two different conformations of the quadrimers of SMA at pH 3 corresponding to two chiralities (SR RR sr and SR SR SR). The first structure has a steplike conformation and the second a well-like conformation. Reproduced with permission from [S1]. Figure S3: Front view and side view of the configuration of the tubular association of SMA dodecamers at pH 7 at the molecular mechanical level. Reproduced with permission from [S3], Table S1: Comparison of the inner and outer diameter of the tubular structure of SMA at pH7 obtained from ab initio molecular modelling and neutron scattering. Reproduced with permission from [S2], Figure S4. DLS results of (a) pure 0.05 wt % SMA solution (reprinted with permission from [S4]) (b) 1 wt % PSMA-DABA-FA with mean zeta potential of −39.89 mV. The presented line in the graph is a guide for the eyes. Reproduced with permission from [S4], Figure S5: FA-DABA-PSMA oligomer at pH 7 at #1 carboxylic acid using ONIOM Model: central trimer optimized to DFT, outer two to PM6. Reproduced with permission from [S4], Figure S6: 2,4-DABA variant folate-conjugated PSMA quadrimer bonded at the #2 carboxylic acid at pH 3. Each monomer forms a ~90° angle with its neighbour. Reproduced with permission from [S4].

## Abbreviations

5-FU	fluorouracil
Cur	curcumin
DABA	2,4-diaminobutyric acid
ECM	extracellular matrix
EPR	enhanced permeability and retention
FA-SMA	folate functionalized SMA
FRα	folate acid receptor alpha
MCTS	multicellular tumour spheroid(s)
MDA-MB231	human triple negative breast cancer cell line
NRP1	neuropilin-1
PANC-1	human pancreatic cancer cell line
PEG	polyethylene glycol
pH_e_	extracellular pH
RGDfK	Arg-Gly-Asp-D-Phe-Lys
SMA	poly(styrene-*alt*-maleic anhydride)
TPP	triphenyl phosphonium cation
Wt	weight
