# Supplementary material for: Functionalized Folic Acid-Conjugated Amphiphilic Alternating Copolymer Actively Targets 3D Multicellular Tumour Spheroids and Delivers the Hydrophobic Drug to the Inner Core
Source: Nanomaterials (Basel). 2018 Aug 2;8(8):588. doi: 10.3390/nano8080588 (PMC6116205; doi:10.3390/nano8080588)
Supplement: Supplementary file 1 [file nanomaterials-08-00588-s001.pdf]

Supplementary Data on the Characterization of the Nanopolymer

The data presented in the supplementary information represent a summary of the characterization of the self-assembly of both the un-functionalized and functionalized polymers self-assembly as a function of pH. The characterization was presented in detailed in the following published articles:

The change in linearity of the backbone of the un-functionalized polymer from pH7 to pH3 explains the disruption of the self-assembly process at low pH (Figure S1 and Figure S2). The tubular structure is only observed at pH7 (Figure S3). The same structural change was characterized by Small Angle Neutron Scattering [S2] with a very close agreement between predicted values by molecular modelling and experimental values for the specificities of the self-assembled nanostructure (Table S1).

The change in hydrodynamic radius was also used as a marker for the self-assembly during this study and was also used to characterize the pH responsiveness of the functionalized polymer as reflected in Figure S4. The structural change between pH7 and pH3 was also modelled and confirmed the pH responsiveness of the functionalized polymer (Figures S5 and S6) as well as its great potential as a targeted drug delivery carrier for cancer treatment [S4].

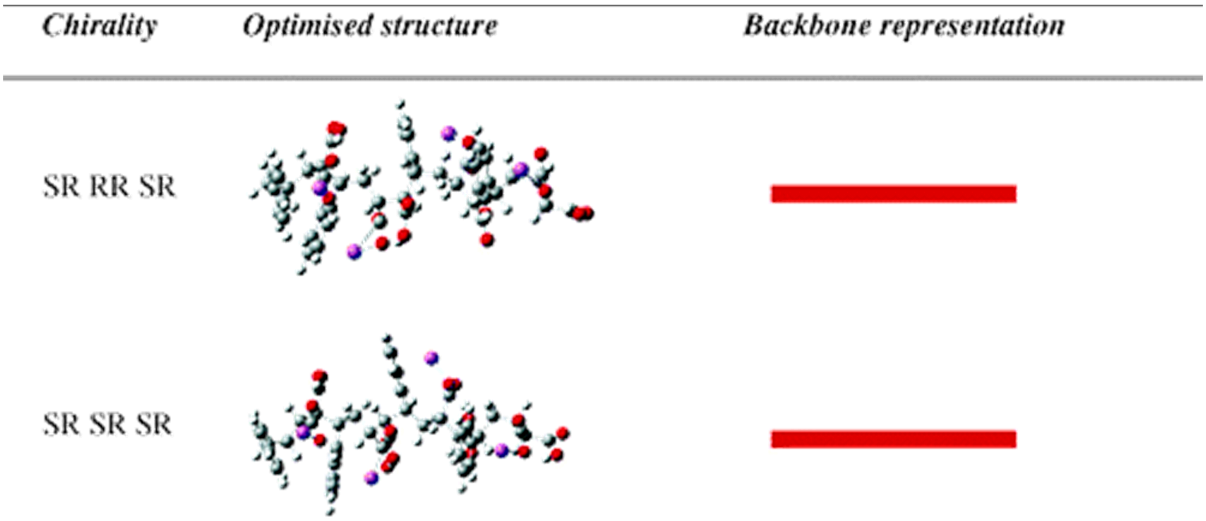

Figure S1. Two different conformations of the quadrimers of SMA at pH 7 corresponding to two chiralities (SR–RR–SR and SR–SR–SR). The structures are very linear, and the orientations of the benzene groups are similar. Reproduced with permission from [S1].

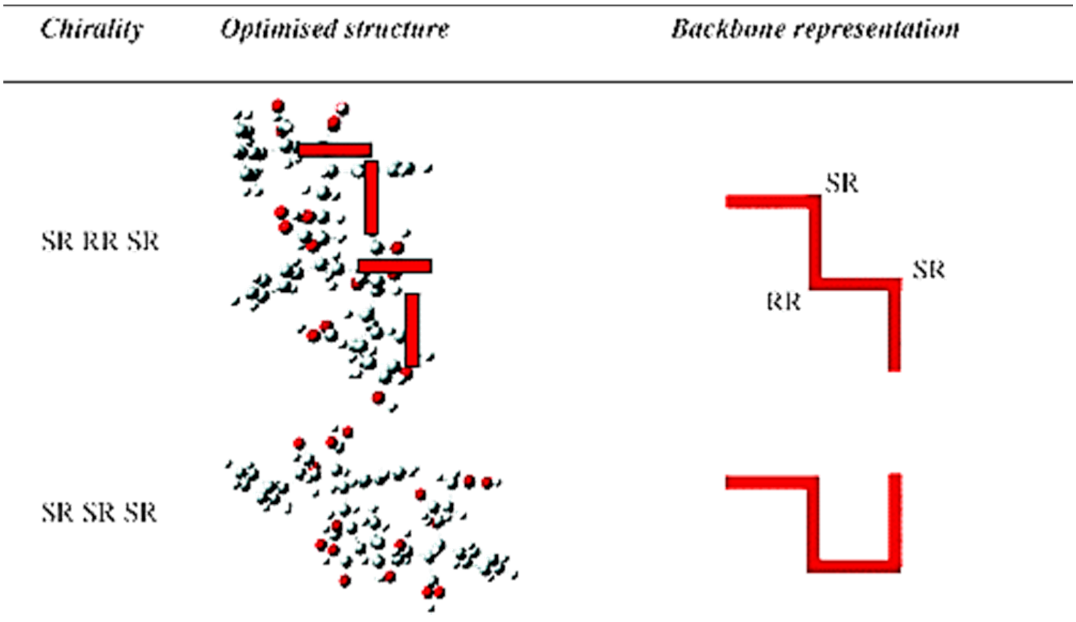

Figure S2. Two different conformations of the quadrimers of SMA at pH 3 corresponding to two chiralities (SR RR sr and SR SR SR). The first structure has a steplike conformation and the second a well-like conformation. Reproduced with permission from [S1].

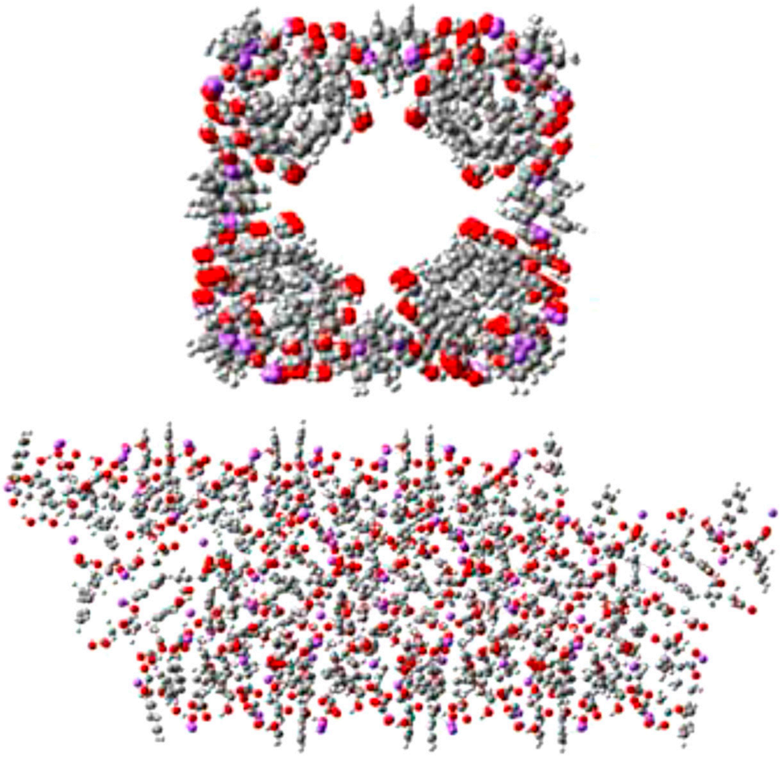

Figure S3. Front view and side view of the configuration of the tubular association of SMA dodecamers at pH 7 at the molecular mechanical level. Reproduced with permission from [S3].

Table S1. Comparison of the inner and outer diameter of the tubular structure of SMA at pH7 obtained from ab initio molecular modelling and neutron scattering. Reproduced with permission from [S2].

	**inner radius (Å)**		**outer radius (Å)**	
	theory	experiment	theory	experiment
0.5 wt %	14.0	13.0 ± 0.8	20.5	19.9 ± 1.5
1 wt %	14.0	12.8 ± 0.6	20.5	19.7 ± 1.2
2 wt %	14.0	12.8 ± 0.6	20.5	22.3 ± 0.7
5 wt %	14.0	12.7 ± 0.7	20.5	22.3 ± 0.7
10 wt %	14.0	12.0 ± 1.4	20.5	21.9 ± 1.0

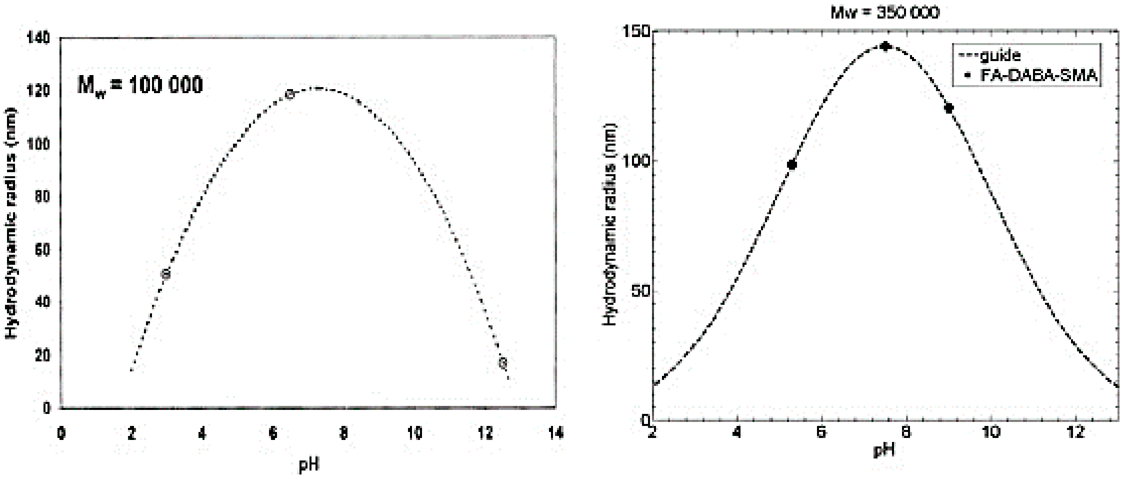

Figure S4. DLS results of a) pure 0.05 wt% SMA solution (reprinted with permission from [44]) b) 1 wt% PSMA-DABA-FA with mean zeta potential of − 39.89 mV. The presented line in the graph is a guide for the eyes. Reproduced with permission from [S4].

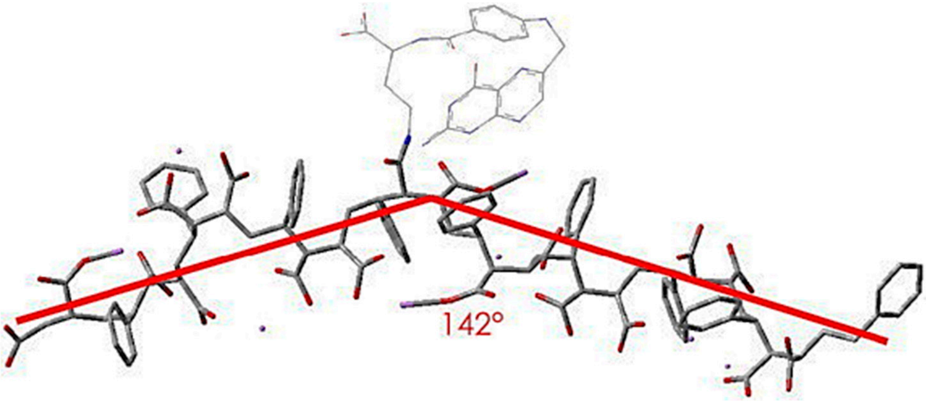

Figure S5. FA-DABA-PSMA oligomer at pH 7 at #1 carboxylic acid using ONIOM Model: central trimer optimized to DFT, outer two to PM6. Reproduced with permission from [S4].

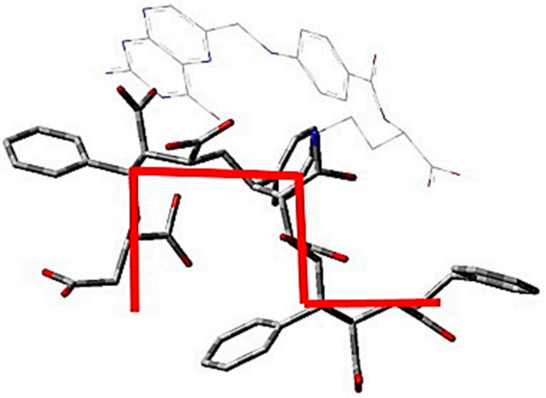

Figure S6. 2,4-DABA variant folate-conjugated PSMA quadrimer bonded at the #2 carboxylic acid at pH 3. Each monomer forms a ~ 90° angle with its neighbour. Reproduced with permission from [S4].

[S1] "Linear conformation of poly (styrene-a lt-maleic anhydride) capable of self-assembly: a result of chain stiffening by internal hydrogen bonds" C Malardier-Jugroot, TGM van de Ven, MA Whitehead, The Journal of Physical Chemistry B 109 (15), 7022-7032, 2005

[S2] "Novel self-assembly of amphiphilic copolymers into nanotubes: characterization by small-angle neutron scattering" C Malardier-Jugroot, et al., Langmuir 21 (22), 10179-10187, 2005

[S3] "Characterization of a novel self-association of an alternating copolymer into nanotubes in solution" C Malardier-Jugroot, TGM Van de Ven, MA Whitehead, Molecular Simulation 31 (2-3), 173-178, 2005

[S4] "Synthesis and characterization of a pH responsive folic acid functionalized polymeric drug delivery system" X Li, M McTaggart, C Malardier-Jugroot, Biophysical chemistry 214, 17–26, 2016

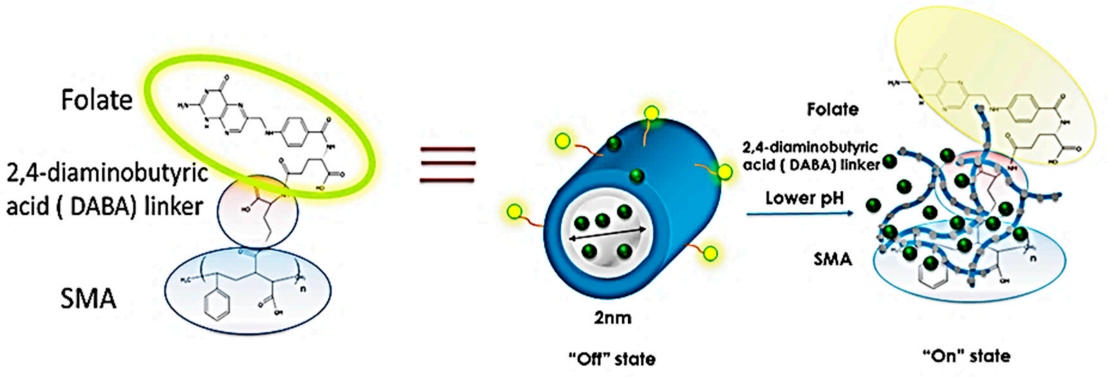

Graphical Abstract: This schematic illustrates a pH-responsive, "smart" active polymeric delivery system using folate (green) functionalized amphiphilic alternating copolymer poly(styrene-*alt*-maleic anhydride) (FA-DABA-SMA) via a biodegradable linker (red) 2,4-diaminobutyric acid (DABA). The "off state" occurs at a neutral pH when the polymer self-assembles into an ordered nanotube conformation and is capable of encapsulating a hydrophobic agent (green). In an acidic tumour microenvironment, these nanotubes change their conformation into individual chains thereby effectively releasing their cargo. Taken in part from Sambi M, Qorri B, Malardier-Jugroot C, Szewczuk MR (2017) Advancements in Polymer Science: 'Smart' Drug Delivery Systems for the Treatment of Cancer. MOJ Poly Sci 1(3): 00016. DOI: 10.15406/mojps.2017.01.00016. Publisher and licensee MedCrave Group. This is an Open Access article which permits unrestricted non-commercial use, provided the original work is properly cited.
